# A Detection-Service-Mobile Three-Terminal Software Platform for Point-of-Care Infectious Disease Detection System

**DOI:** 10.3390/bios12090684

**Published:** 2022-08-25

**Authors:** Xiangyi Su, Yile Fang, Haoran Liu, Yue Wang, Minjie Ji, Zhu Chen, Hui Chen, Song Li, Yan Deng, Lian Jin, Yuanying Zhang, Murugan Ramalingam, Nongyue He

**Affiliations:** 1 State Key Laboratory of Bioelectronics, School of Biological Science and Medical Engineering, Southeast University, Nanjing 210096, China; 2 Hunan Key Laboratory of Biomedical Nanomaterials and Devices, Hunan University of Technology, Zhuzhou 412007, China; 3 Department of Molecular Biology, Jiangsu Cancer Hospital, Nanjing 210009, China; 4 Institute of Tissue Regeneration Engineering, Department of Nanobiomedical Science, BK21 NBM Global Research Center for Regenerative Medicine, Dankook University, Cheonan 31116, Korea; 5 School of Basic Medical Sciences, Chengdu University, Chengdu 610106, China

**Keywords:** point-of-care testing, three-terminal software, geographic information system, infectious disease warning, machine learning

## Abstract

The traditional infectious disease detection process is cumbersome, and there is only a single application scenario. In recent years, with the development of the medical industry and the impact of the epidemic situation, the number of infectious disease detection instruments based on nursing point detection has been increasing. Due to this trend, many detection instruments and massive detection data urgently need to be managed. In addition, the experiment failed due to the abnormal fluorescence curve generated by a human operator or sample impurities. Finally, the geographic information system has also played an active role in spreading and preventing infectious diseases; this paper designs a “detection-service-mobile” three-terminal system to realize the control of diagnostic instruments and the comprehensive management of data. Machine learning is used to classify the enlarged curve and calculate the cycle threshold of the positive curve; combined with a geographic information system, the detection results are marked on the mobile terminal map to realize the visual display of the positive results of nucleic acid amplification detection and the early warning of infectious diseases. In the research, applying this system to portable field pathogen detection is feasible and practical.

## 1. Introduction

Common strategies for clinical diagnosis of infectious diseases depend on: (1) culture, identification, or observation of pathogens [1,2,3,4]; (2) detection of specific antigens and antibodies of pathogens [5,6,7]; (3) enzyme-linked immunosorbent assay (ELISA) [8]; (4) systematic evolution of ligands by exponential enrichment (SELEX) [9,10,11]; (5) nucleic acid testing (NAT) [12,13,14,15]. These approaches often rely on specialized medical facilities and the testing process is cumbersome, requiring professionals to operate special testing instruments to complete the relevant testing. Point-of-care testing (POCT) refers to clinical and bedside tests conducted near patients, which usually do not need to be undertaken by clinical doctors. Instruments based on POCT [16,17,18,19] overcome the limitations of dedicated laboratories, and can meet the needs for timely and local testing in hospital emergencies, epidemic sites, port quarantine, and other scenarios in regards to grassroots health institutions. With the development of POCT, the number of in vitro diagnostic instruments and testing data has also increased [20,21,22]. However, the data management mode of traditional in vitro diagnostic instruments has not entirely separated from the manual state, affecting the staff’s work efficiency. It is urgent to develop a data management platform based on infectious disease detection to provide laboratory personnel with an intelligent, paperless, standardized working environment. In addition, abnormal curves can be generated due to human operation, hardware interference, or sample impurities, resulting in experimental failure. Traditional manual review of nucleic acid amplification curves is time-consuming and laborious. It is only suitable for small batch inspection, cannot process massive data, and is affected by human judgment [23]. Therefore, there is an urgent need for application software that can automatically interpret the cause classification of abnormal results for users. Machine learning can solve classification problems, and many researchers have used it to classify physiological signals [24,25,26]. Finally, geoinformation technology has played an active role in transmitting and preventing infectious diseases, providing a basis for epidemic prevention, control, and management [27,28,29].

This paper expounds and summarizes some existing relevant and excellent network platforms. The LinRegPCR application [30] for the web developed by Andreas Untergasse enables the running of quantitative real-time polymerase chain reaction (qPCR) experiments and visual analysis of data, showing the results of amplification curve analysis and melting curve analysis in tables and graphs. In the early stage, our research group [31] developed a software control system suitable for on-site pathogen detection equipment. Based on small nucleic acid detection equipment, this system studied a software system that could realize on-site automatic detection and data cloud storage. The system collects the data of the qPCR instrument through the computer, performs preprocessing, fitting, abnormal analysis, cycle threshold (Ct) value solution, and other operations on the data, and can upload the data for online analysis through the web end.

The network platform of the infectious disease detection instrument constructed by the above work only includes the detection and service end. The data of the detection instrument requires the user to manually input the website on the browser for viewing, which is not convenient enough. Previous work has not classified the abnormal fluorescence amplification curve, or the analysis is not comprehensive enough. There is no exploration of the early warning of infectious diseases by using the geographical location information of detection instruments. Given this situation, this paper develops a detection-service-mobile three-terminal software program for a portable on-site pathogen detection system independently researched by our research group. The detection and the mobile terminals are based on the Android system and are integrated with the Baidu map software development kit (SDK), and the service end uses the Django framework. This paper has achieved the following:

1. The automatic process control of “sample in, result out” can classify the fluorescence amplification curve to realize abnormal curve recognition, calculate the Ct value of the positive curve, and generate the detection report.
2. We solved the problem of real-time collection, sharing, management, and analysis of the data generated by the system.
3. With mobile internet, database and geographic information system (GIS) technology provides users with an infectious disease distribution map display, early warning, and other functions.

In the remaining part of this paper, we first briefly introduce the overall architecture and workflow of the three-terminal system built in this paper. Then, the flow of fluorescence curve analysis algorithm is introduced in detail. The basis of selecting the classifier, the process of training the classifier, and the method of solving the Ct value are described, respectively. The next part presents the user interface of the detection and mobile software, the evaluation results of the classifier, and the experimental results when comparing the proposed Ct algorithm with commercial instruments. Finally, the full text is summarized. In addition, limitations and areas for improvement are described.

## 2. Materials and Methods

### 2.1. Architecture and Workflow of the System

**Figure 1:**
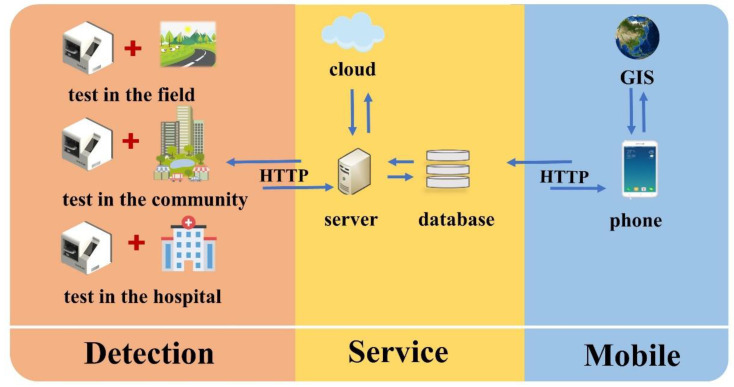
The overall architecture of the three-terminal software. The detection terminal can be used in different scenarios, and the data can be synchronized to the server so that the mobile terminal can view the data and address the location of the instrument anytime and anywhere and display this visually.

This paper proposes the three-terminal software architecture, which is the detection-service-mobile. The overall architecture of the three-terminal software is shown in Figure 1. The functions of each part are described below.

The detection software is based on the Android system, and a touch screen embedded in the instrument is used as the carrier, which realizes the integration of the instrument and control software, solves the pain point of space limitation of large-scale medical equipment, and causes the application scenarios of in vitro diagnosis to develop in a diversified direction. Rapid detection can be conducted in hospitals, communities, outdoors, and in families. It is developed on Android Studio by Java. Model–View–Control (MVC) [32] software design model separates business logic, data, and interface display. The principle of “high cohesion, low coupling” is divided into modular design and implementation. On the one hand, the detection terminal sends instructions to control the operation of the lower machine through the controller area network (CAN) protocol to realize the automatic operation of nucleic acid extraction, qPCR reaction, and fluorescence detection. On the other hand, the detection end integrates the Baidu map SDK to realize the geographic information positioning of the instrument. At the same time, when the network is available, the testing end collaborates with the server to complete user login, synchronous update, data upload, and other operations during the instrument use.

The server uses the Django framework and MySQL database to manage detection instruments and detection data. At the same time, the server is deployed to the cloud to realize the “cloud storage” of all kinds of data and facilitate the mobile terminal to access data anytime and anywhere.

The mobile terminal is based on Android and uses the mobile phone as the carrier to access server data through the hypertext transfer protocol (HTTP). The mobile terminal also integrates the Baidu map SDK, which can mark positive results on the map. It breaks down the number of positive results detected by each instrument into four risk levels and visualizes them using different colors on a map. Users can use the system to accurately and quickly access the epidemic information on infectious diseases and can play a role in monitoring infectious diseases.

**Figure 2:**
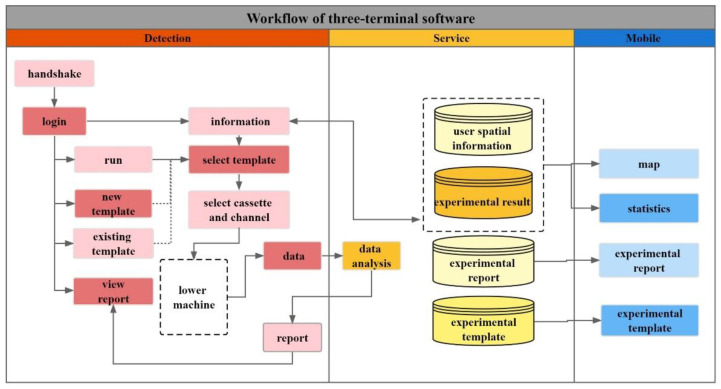
Workflow of three-terminal software. Describes the workflow when the user uses the software to realize the automatic control of the experiment. First, the detection terminal obtains data by controlling the automatic experiment of the lower computer and then synchronizes the data to the server. Finally, the mobile terminal accesses the database and displays the results on the map.

The workflow when users use this software to realize the automatic control of the experiment is illustrated in Figure 2. When the detection end is running, it will perform a self-test command and shake hands with the lower machine. After the handshake is successful, it will jump to the main page of the detection end for user login. When there is a network, the server will obtain the user’s basic information and geographical location and store the data in the user’s spatial information database. After successful login, the user can delete, modify, and view the template routinely. The template data are stored in an extensible markup language (XML) format, and the file can be uploaded to the detection template database on the server. The user can choose to use the self-edited template or the default template, select cassettes and channels to experiment with, and finally click the run button to start the experiment. The animation is used to indicate the progress of experiments, such as nucleic acid extraction, qPCR amplification, and dynamic generation of fluorescence amplification curves. After the experiment is completed, the detection end will immediately analyze the fluorescence data and generate the experimental report in the hypertext markup language (HTML) format. The report includes current CT, experimental results, and fluorescence amplification curves. Accordingly, the server will also obtain and store the experimental report in the experimental report database. Accordingly, the server will also obtain a synchronous experiment template and report stored in the database, along with the user’s location and the experimental results to generate a new data table. When the mobile terminal accesses the server, the server will be accessed according to the geographical location of the spatial database of the user; the positive cases are marked on the map at the location of the information.

### 2.2. Fluorescence Data Analysis

**Figure 3:**
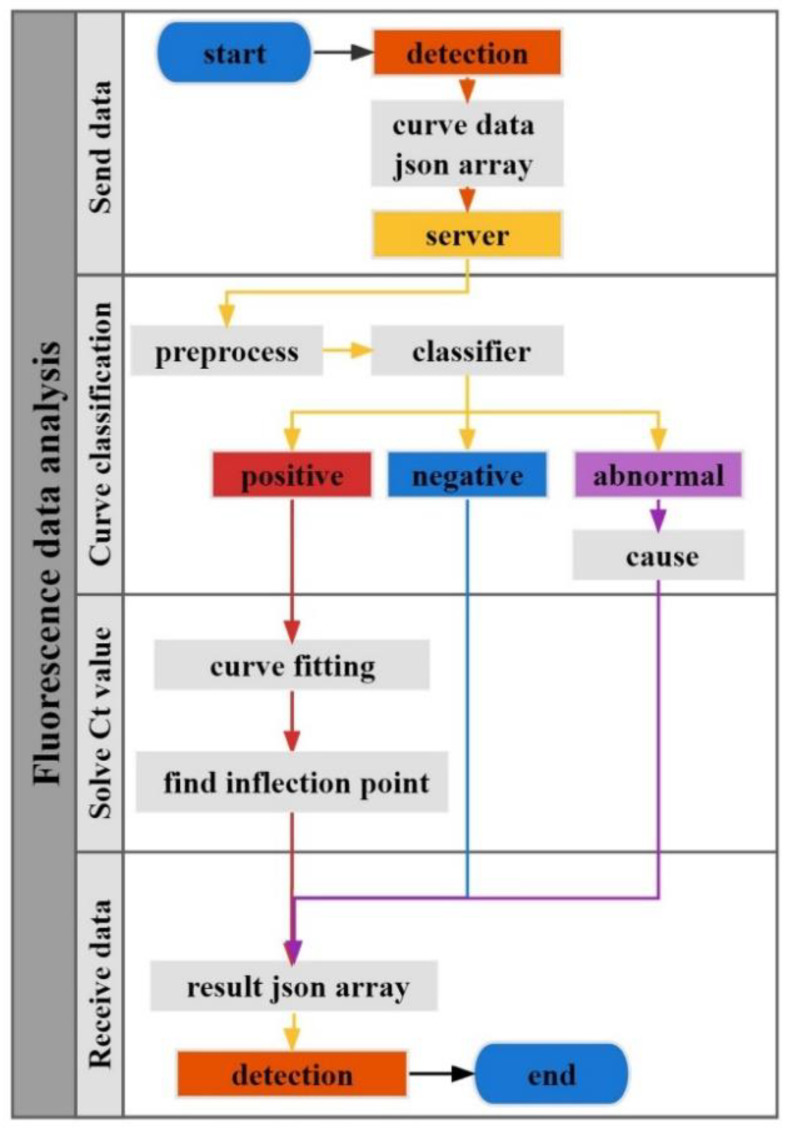
Flow chart of fluorescence curve analysis. It is divided into four parts: send data, curve classification, Ct value calculation, and receive data.

Fluorescence amplification detection refers to adding fluorophores to the nucleic acid amplification reaction system and the real-time monitoring of the whole amplification process by accumulating fluorescence signals. The flow chart of fluorescence curve analysis is shown in Figure 3. After receiving the data sent by the detection end, the server first normalizes the data and then inputs the data into the classifier for curve classification, which is mainly divided into three categories: positive, negative, and abnormal. For the positive curve, one must solve the Ct value. For the abnormal curve, one must find the corresponding reason according to the preset abnormal curve type. Finally, the curve analysis results are integrated into an array and returned to the detection end.

**Figure 4:**
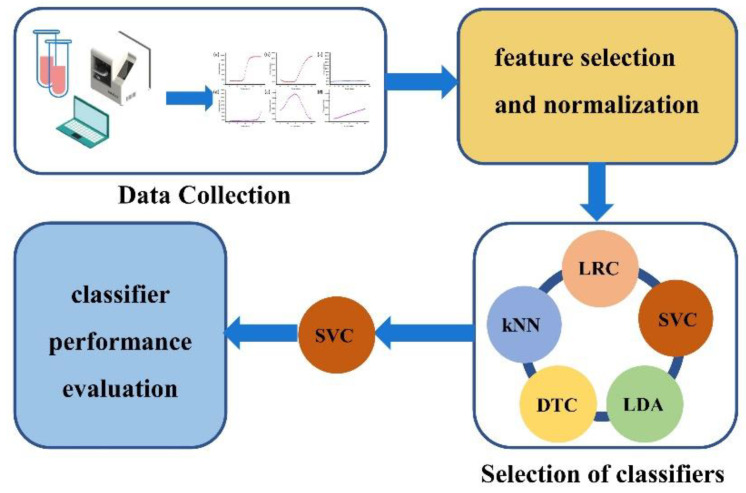
The steps of classifier selection and training. It includes data collection, feature selection and normalization, classifier selection, and performance evaluation.

Figure 4 illustrates the steps of classifier selection and training, including data set creation, preprocessing and feature extraction, and classifier selection including Supporting Vector Classifier (SVC), Logistic Regression Classifier (LRC), k-Nearest Neighbors (kNN), Decision Tree Classifier (DTC), and Linear Discriminant Analysis (LDA), along with performance evaluation. In this study, the Scikit-Learn tool kit in Python is used to achieve multiple classifications of curves.

#### 2.2.1. Data Collection

**Figure 5:**
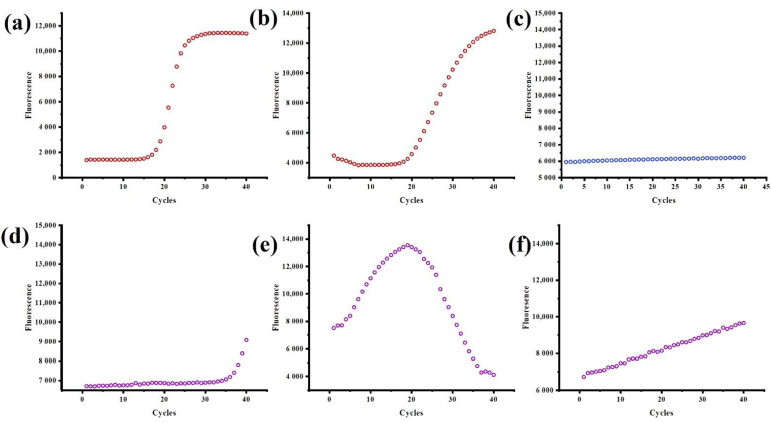
Six types of curves are input to the classifier. Different curve types are distinguished by the color of dot. Positive curve, negative curve and abnormal curve use red dot, blue dot and purple dot respectively (**a**) The curve was s-shaped, with an obvious exponential phase and plateau phase. (**b**) The rising rate was slow at the peak stage of the amplification phase and fast at the later stage of the amplification phase. (**c**) Straight line parallel to the X axis. (**d**) Jump at the end of the curve. (**e**) The curve showed a trend of rising first and then declining. (**f**) Upward drift line or approximate upward drift line.

**Figure 6:**
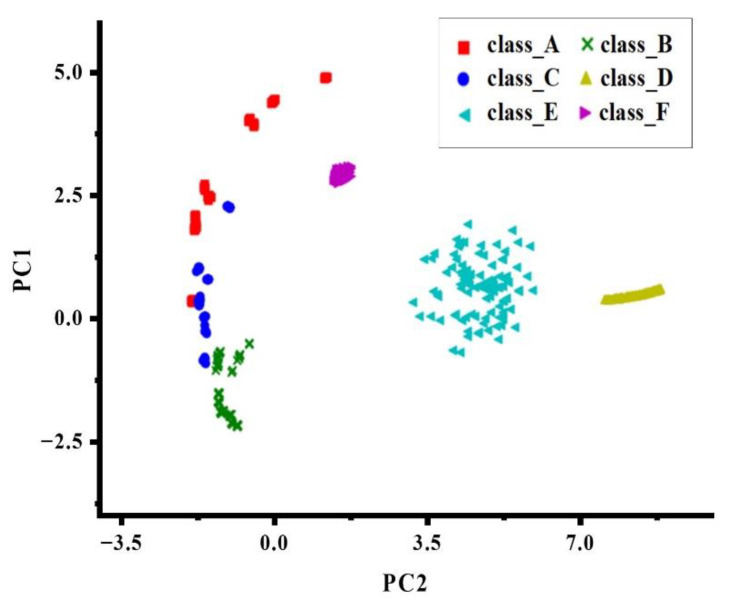
Principal component analysis diagram of six curves.

Because machine learning needs to use a large number of data sets for training, and the acquisition of fluorescence data is not easy, this paper simulates six types of data in Figure 5 based on the actual data obtained by the nucleic acid detection instrument developed by our research group. Spreadsheet S1 contains simulated PCR amplification curve data used as a dataset for machine learning training classifiers. The causes of the curve anomalies are explained in Table 1. Each sample is a 1 * 41 vector, including 1 label and 40 features. Each type has 100 samples, a total of 600 pieces of data. Figure 6 is the principal component analysis diagram after reducing the 40 features to 2 dimensions. It can be seen that all six types have good discrimination.

#### 2.2.2. Feature Selection and Normalization

The characteristic value of the sample is the fluorescence value obtained at the end of each qPCR cycle. Before the samples are input into the classifier, we need to normalize the data to solve the comparability between the data indicators. After data standardization, all indexes of original data are in the same order of magnitude, which is suitable for comprehensive comparative evaluation.

#### 2.2.3. Selection of Classifiers

The classifier is trained by dividing the training set and the test set into 7:3. We also use the gridsearchcv function in sklearn to cross-validate the grid search and choose a 5-fold cross-validation scheme to calculate the evaluation score. This method can traverse all possibilities and determine the best hyper-parameters of all the above machine learning algorithms in a short time.

#### 2.2.4. Performance Evaluation

The confusion matrix is a standard performance evaluation index in multi-classification problems, through which we can intuitively see the specific results of the accurate and inaccurate model prediction. The elements on the main diagonal of the confusion matrix correspond to the correct classification, while other elements tell us how many samples in one category are incorrectly classified into other categories. The confusion matrix shows which part of the classification model will be confused when making predictions. This decomposition of the results overcomes the limitations of using only the classification accuracy.

#### 2.2.5. Positive Curve Fitting and Ct Value Solution

For the positive curve, we use the Gauss–Newton iterative method to fit the original data to the 5-parameter logistic model to obtain the specific functional form. Then, we solve the Ct value, that is, the abscissa of the maximum value of the second derivative of the fitting curve. The advantage of this method is that it automatically calculates the Ct value through the algorithm to avoid introducing errors into the subjective selection data.

#### 2.2.6. Algorithm Verification Experiment

In order to verify the accuracy of the algorithm for solving the Ct value in this paper, the algorithm verification experiment was carried out. The Ct value obtained by the algorithm in this paper is compared with the result obtained by the commercial qPCR instrument. The specific steps are as follows:

1. The qPCR experiment was performed in StepOnePlus^TM^ Real-Time PCR Systems of Applied Biosystems. The instrument application software is StepOne^TM^ software (version 2.3). The virus used is hepatitis B virus (HBV) nucleic acid assay kit (Z-HD-2002-02, Shanghai Zhijiang Biotechnology Co., Ltd.) with standard serum samples from the kit with HBV concentration of 5 × 10^3^ IU/mL~5 × 10^8^ IU/mL.
2. After the experiment, we exported the original data of the StepOne^TM^ software. The raw data is shown in Spreadsheets S2.
3. We used the instrument’s original fluorescence data and this paper’s algorithm program to calculate the Ct value.
4. We sorted out the results and performed a comparative analysis.

## 3. Results

### 3.1. User Interface 

#### 3.1.1. The User Interface of the Detection Software

**Figure 7:**
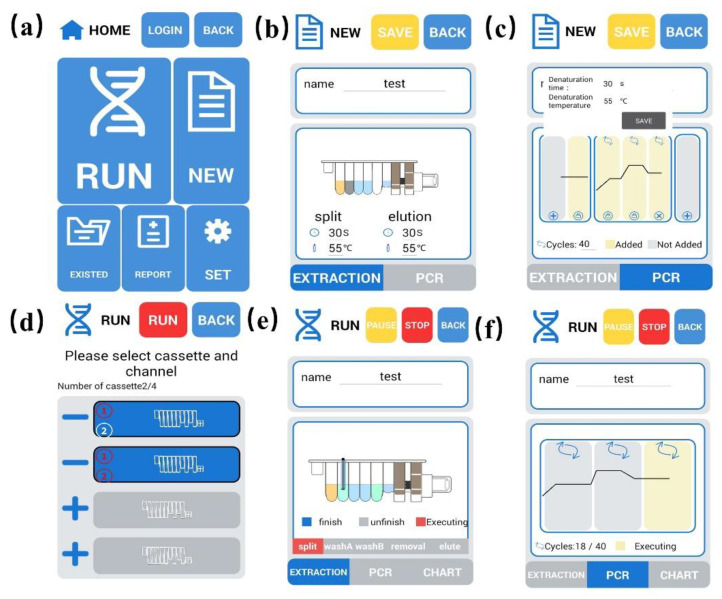
The main interface of the detection software. (**a**) The main page, (**b**) the configuration page of nucleic acid extraction parameters, (**c**) the qPCR parameter configuration page, (**d**) the page for selecting card boxes and channels, (**e**) the page for nucleic acid extraction, and (**f**) the page for qPCR.

After using the prototype design platform to design the interface and specific functions, we then used Java to design the software and used Photoshop to make materials needed by the software. The user interface of the detection software is shown in Figure 7, which was achieved: Figure 7a is the main page. There are six modules on the main page: running, new template, existing template, report, and log in. The main page is the function selection entrance of the full software. Figure 7b,c show the new template page, where the parameters of the experiment template are configured. Figure 7b illustrates the configuration page of nucleic acid extraction parameters, mainly configuring the time and temperature of nucleic acid lysis and elution. The qPCR parameter configuration page in the new template mainly configures the time and temperature of each qPCR step (such as hot start, pre-denaturation, denaturation, etc.); the number of qPCR cycles or some steps are added or not added according to the specific experiment, as shown in Figure 7c. In addition, some fixed parameters on the settings page will not be covered here. Figure 7d shows the page for selecting card boxes and channels before the experiment. There are altogether four card boxes, and each card box has two channels to choose from. All channels use the same template for the experiment at the same time. Figure 7e shows the page on nucleic acid extraction. There are six steps in nucleic acid extraction by magnetic beads, including cracking, washing A, washing B, alcohol removal, and elution. Each step has three states, done, unfinished, and running, and uses the graphics interchange format (GIF) to indicate the run phase. Figure 7f is the page for qPCR, showing the current number of qPCR cycles and the current stage.

#### 3.1.2. The User Interface of the Mobile Software

**Figure 8:**
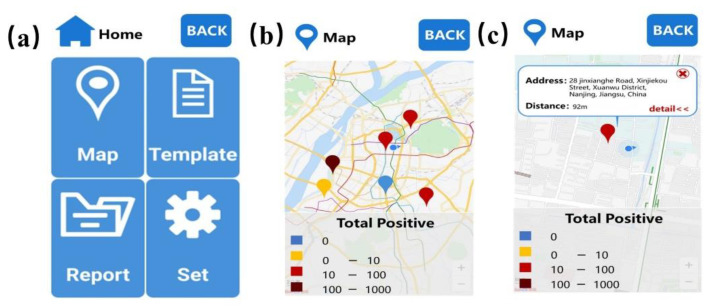
The main interface of the mobile software. (**a**) Home page of the mobile software. (**b**,**c**) Map visualization.

The user interface of the mobile software is shown in Figure 8. Figure 8a is the home page of the mobile terminal software, on which there are four major modules, namely, map, template, report, and setting. Map visualization and early warning of infectious diseases are mainly related to the map module. Figure 8b, c visualize the map. The position of the instrument using the detection software will be marked on the map, and the color of the mark is related to the total number of positive results detected by the instrument. One must click the annotation to view the instrument and apparatus positioning information and the distance between the user locations.

### 3.2. The Result of Fluorescence Amplification Curve Analysis

#### 3.2.1. Classifier Selection and Performance Evaluation

We used precision, recall, accuracy, and f1 indicators to select classifiers. Table 2 shows that all five algorithms have good results. The highest score among the four parameters is SVC. It has two outstanding advantages compared to other statistical prediction models or learning algorithms: (1) it has a well-researched core technology that can deal with linear inseparable problems. (2) Combined with optimization theory, statistics, and function analysis, it has high computational efficiency and strong prediction ability and has advantages in solving minor sample problems. Later, we determined SVC as the final classification model. The optimized parameters are C = 0.5, kernel = RBF, and Gamma = 1. 

**Figure 9:**
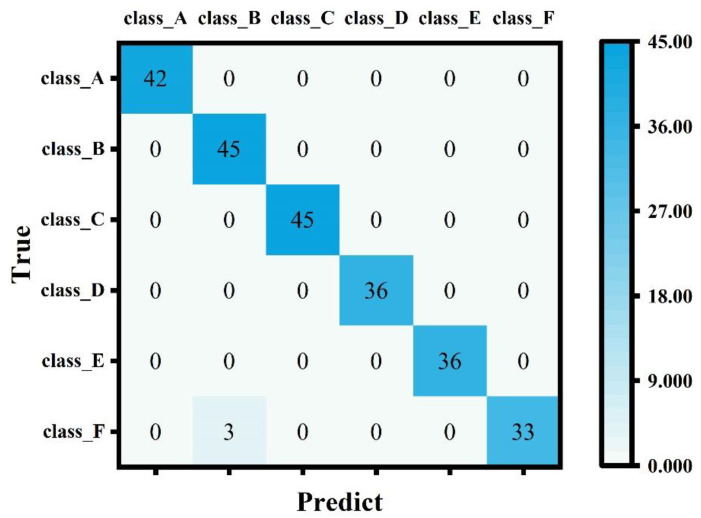
Confusion matrix of SVC in the form of the heat map. The larger the value, the darker the color, which can help us clearly see the prediction effect of each category.

The heat map form of the confusion matrix of SVC is shown in Figure 9. The sum of each row represents the true number of samples in that category, and the sum of each column represents the number of samples predicted to be in that category. We can see that most of the non-zero values are on the diagonal, which means that the classification is correct. The 3 in the second column of the first row indicates that the three instances that belong to the first category are incorrectly predicted as the second category, which indicates that there is still room for improvement in the classifier’s ability to distinguish class_A from class_B.

Machine learning technology is an application of artificial intelligence used in different areas of human research. We used the SVC for nucleic acid detection to classify fluorescence amplification curves. Many researchers have used different methods to classify fluorescence amplification curves. Compared with previous studies, our analysis saves human resources for discrimination, classifies curves in more detail, and achieves maximum accuracy. The comparison results are given in Table 3.

#### 3.2.2. The Solution Results of the Ct Values

**Figure 10:**
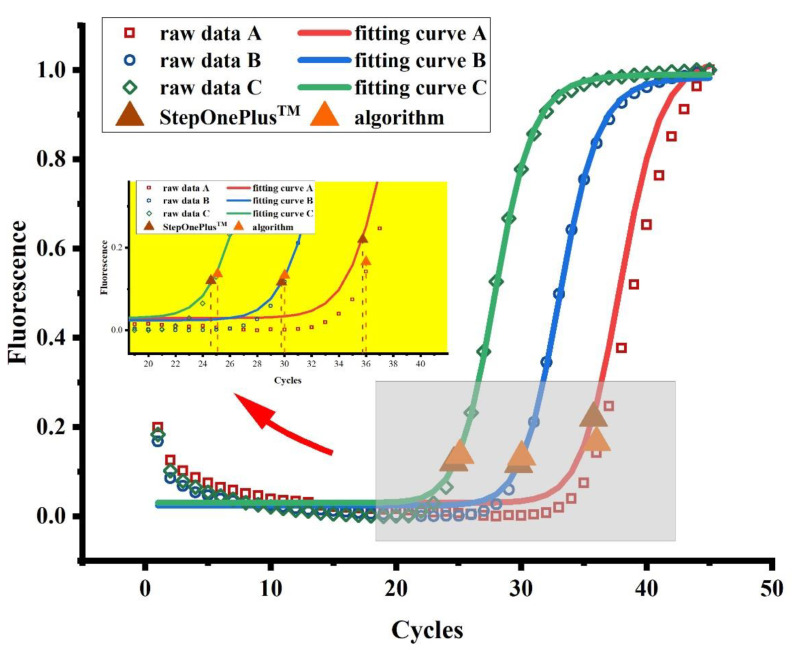
Curve fitting diagram of original data. The original data and fitted curves of HBV samples at three different concentrations are displayed in a graph, and the amplification area is enlarged. In the figure, the positions of inflection points calculated by StepOnePlus^TM^ and the self-developed algorithm are marked, respectively. The abscissa of the inflection point is the Ct value. The specific values are shown in Table 4.

The Ct values obtained using the algorithm proposed in this paper and the commercial instrument are shown in Table 4 (two decimal places are reserved). We show the original data of samples A, B, and C in Figure 10. The amplification area of the curve is enlarged to compare the Ct values of the two methods. We used the Pearsonr module in Python to show that there are no significant differences between the two methods. The Pearsonr correlation coefficient of the two data groups in Table 4 is 0.99, and the significance value is 0.0009. That is to say, the two methods have a strong correlation and can replace each other, which verifies that the self-developed algorithm is effective.

## 4. Discussion

Based on the research in this paper, the following aspects will be further optimized and improved in the subsequent platform software research and development of the project, combined with the actual application feedback of on-site pathogen detection. First, the qPCR amplification curve data collected by the detection terminal are stored in the database, not in accordance with the international standard storage, which is not conducive to the sharing and exchange of experimental data. The storage format will be modified by referring to minimum information for later publication of quantitative real-time qPCR experiments (MIQE) [33]. Secondly, SVC was used to classify qPCR data with different morphologies and implement the cause of abnormal inference. However, this process has many limitations. Data are trained classifier simulation data; the lack of actual experimental data and the abnormal discriminant cannot be realistic, and the abnormal curve classification also needs to develop in the experiment. In addition, since the classification model is deployed on the server, the entire curve analysis process is constrained by the network. Finally, for the early warning of infectious diseases on mobile terminals, the current work serves only to combine the geographical location of the instrument with the database to display the statistical results of multiple dimensions visually; the relationship between geographical location and epidemic spread will be explored in the future.

## 5. Conclusions

This paper designs and implements a portable three-terminal system for field pathogen detection for practical problems and completes the whole software development process, including software requirements analysis, framework design, code implementation, software testing, and verification. We completed the integrated nucleic acid detector’s software system design and the data storage, management, display, analysis, and report generation functions. We improved the integration, automation, intelligence, and simplicity of operation of the instrument. According to the qPCR curve analysis of the instrument, the machine learning method is used to classify the qPCR data with different shapes, and the Ct value of the positive curve is calculated. In addition, the detection results are combined with the geographic information system to form a spatiotemporal database of an epidemic situation with spatiotemporal information characteristics, and the positive results are marked on the map, which provides strong support for the visual display of the positive results of nucleic acid amplification detection and the timely early warning of infectious diseases. The experiment proves that the system is feasible and practical when used in a portable field pathogen detection system.

## Figures and Tables

**Table 1:** Abnormal causes corresponding to six kinds of curves.

Type	Curve	Type	Operation	Abnormal Cause [23]
class_A	Figure 5a	positive	Ct value calculation	——
class_B	Figure 5b			
class_C	Figure 5c	negative	——	——
class_D	Figure 5d	abnormal	warning abnormal cause	suspected cross-contamination of the template or low sample concentration
class_E	Figure 5e			severe evaporation of samples or probe degradation
class_F	Figure 5f			slight sample evaporation

**Table 2:** Comparison of five classifiers on the accuracy, recall, precision and f1 scores.

Classifier	Precision	Recall	Accuracy	F1
SVC	0.99	0.99	0.99	0.99
LRC	0.97	0.97	0.96	0.96
kNN	0.99	0.99	0.98	0.98
DTC	0.96	0.95	0.96	0.96
LDA	0.93	0.92	0.92	0.92

**Table 3:** Comparison of the accuracy of curve classification between the previous research and this article.

Reference	Year	Sample	Method	Accuracy
Chen [23]	2019	fluorescence amplification curve	manual	0.99
Liao [31]	2019	fluorescence amplification curve	machine learning	0.94
this paper	2022	fluorescence amplification curve	machine learning	0.99

**Table 4:** The Ct values calculated by StepOnePlus^TM^ and the self-developed algorithm in this paper.

Method	Sample A	Sample B	Sample C	Sample D	Sample E	Sample F	Sample G	Sample H
StepOnePlus^TM^	35.99	30.12	25.07	34.97	32.41	35.21	34.59	35.92
algorithm	35.70	29.71	24.52	34.53	31.61	34.50	33.96	35.25

## Data Availability

Data are contained within the article.

## Supplementary Materials

The following supporting information can be downloaded at: https://www.mdpi.com/article/10.3390/bios12090684/s1, Spreadsheet S1: Simulated PCR amplification curve data used as a dataset for machine learning training classifiers; Spreadsheet S2: The raw data exported from StepOne^TM^ software.

## References

[1] Gai J., Ma L., Li G., Zhu M., Qiao P., Li X., Zhang H., Zhang Y., Chen Y., Ji W. (2021). A potent neutralizing nanobody against SARS-CoV-2 with inhaled delivery potential. MedComm.

[2] Yang L., Ren L., Tan X., Chu H., Chen J., Zhang Y., Zhou X. (2020). Removal of ofloxacin with biofuel production by oleaginous microalgae Scenedesmus obliquus—ScienceDirect. Bioresour. Technol..

[3] Zhang Y., Ouyang M., Wang H., Zhang B., Guang W., Liu R., Li X., Shih T., Li Z., Cao J. (2020). A cyclic peptide retards the proliferation of DU145 prostate cancer cells in vitro and in vivo through inhibition of FGFR2. MedComm.

[4] Qi J.L., He J.R., Liu C.B., Jin S.M., Gao R.Y., Yang X., Bai H.M., Ma Y.B. (2020). Pulmonary Staphylococcus aureus infection regulates breast cancer cell metastasis via neutrophil extracellular traps (NETs) formation. MedComm.

[5] Liu Y., Li T., Yang G., Deng Y., Mou X., He N. (2022). A simple AuNPs-based colorimetric aptasensor for chlorpyrifos detection. Chin. Chem. Lett..

[6] Guo Z., Liu Y., He N., Deng Y., Jin L. (2020). Discussion of the protein characterization techniques used in the identification of membrane protein targets corresponding to tumor cell aptamers. Chin. Chem. Lett..

[7] Zhao H., Su E., Huang L., Zai Y., Liu Y., Chen Z., Li S., Jin L., He N. (2022). Washing-free chemiluminescence immunoassay for rapid detection of cardiac troponin Ⅰ in whole blood samples. Chin. Chem. Lett..

[8] Höglund K., Palmqvist H., Ringmark S., Svensson A. (2022). Quantification of normetanephrine in canine urine using ELISA: Evaluation of factors affecting results. J. Vet. Diagn. Investig..

[9] Tang Y., Liu H., Chen H., Chen Z., Liu Y., Jin L., Deng Y., Li S., He N. (2020). Advances in Aptamer Screening and Drug Delivery. J. Biomed. Nanotechnol..

[10] Liu Y., Yang G., Li T., Deng Y., Chen Z., He N. (2021). Selection of a DNA aptamer for the development of fluorescent aptasensor for carbaryl detection. Chin. Chem. Lett..

[11] Liu M., Xi L., Tan T., Jin L., Wang Z., He N. (2021). A novel aptamer-based histochemistry assay for specific diagnosis of clinical breast cancer tissues. Chin. Chem. Lett..

[12] He Z., Tong Z., Tan B., He X., Zhang T., Guo Y., Jin L., He N., Li S., Chen Z. (2021). Rapid Detection of DNA Methylation with a Novel Real-Time Fluorescence Recombinase-Aided Amplification Assay. J. Biomed. Nanotechnol..

[13] Li Z., Wang J., Yang H., Chen S., Ma G., Zhang X., Zhu M., Yu J., Singh R., Zhang Y. (2017). Ultrasensitive detection of gastric cancer plasma microRNAs via magnetic beads-based chemiluminescent assay. J. Biomed. Nanotechnol..

[14] Mou X., Sheng D., Chen Z., Liu M., Liu Y., Deng Y., Xu K., Hou R., Zhao J., Zhu Y. (2019). In-situ mutation detection by magnetic beads-probe based on single base extension and its application in genotyping of hepatitis B virus pre-C region 1896nt locus single nucleotide polymorphisms. J. Biomed. Nanotechnol..

[15] Tang Y., Ali Z., Dai J., Liu X., Wu Y., Chen Z., He N., Li S., Wang L. (2018). Single-nucleotide polymorphism genotyping of exoS in pseudomonas aeruginosa using dual-color fluorescence hybridization and magnetic separation. J. Biomed. Nanotechnol..

[16] Chen Z., Yang T., Yang H., Li T., Nie L., Mou X., Deng Y., He N., Li Z., Wang L. (2018). A portable multi-channel turbidity system for rapid detection of pathogens by loop-mediated isothermal amplification. J. Biomed. Nanotechnol..

[17] Xu Y., Wang T., Chen Z., Jin L., Wu Z., Yan J., Zhao X., Cai L., Deng Y., Guo Y. (2021). The point-of-care-testing of nucleic acids by chip, cartridge and paper sensors. Chin. Chem. Lett..

[18] Kuupiel D., Bawontuo V., Mashamba-Thompson T.P.J.D. (2017). Improving the accessibility and efficiency of point-of-care diagnostics Services in low-and Middle-Income Countries: Lean and agile supply chain management. Diagnostics.

[19] Shimetani N.J.M.M.J. (2017). Potential of Next-generation POCT in Infectious Disease Rapid Test. Med. Mycol. J..

[20] Chu S., Wang E., Du L. (2017). Analysis on the Development Trend and Problems of Medical Device Industry in China. Chin. J. Pharm..

[21] Mardanly S.G. (2019). Current state and development trends of domestic medical device market for in vitro diagnostics in the segment of diagnostic reagents and their kits. Klin. Lab. Diagn..

[22] Song H.B., Zhu Y.Y. (2020). The in vitro diagnostics industry in China. View.

[23] Chen X., Liu H., Wang H., Zhao X., Maosheng J.I., Dong Y., Zhang L., Center C.B. (2019). Analysis and treatment of abnormal amplification curve of PCR in blood nucleic acid screening. Chin. J. Blood Transfus..

[24] Prasad P.H., Aakash N.J., Venithraa G., Bala A., Ganesan M., Karthika R. (2020). EEG Signal Analysis Using Machine Learning Techniques. J. Adv. Res. Dyn. Control. Syst..

[25] Bin Heyat M.B., Akhtar F., Khan A., Noor A., Benjdira B., Qamar Y., Abbas S.J., Lai D. (2020). A Novel Hybrid Machine Learning Classification for the Detection of Bruxism Patients Using Physiological Signals. Appl. Sci..

[26] Bin Heyat M.B., Akhtar F., Ansari M.A., Khan A., Alkahtani F., Khan H., Lai D. (2020). Progress in Detection of Insomnia Sleep Disorder: A Comprehensive Review. Curr. Drug Targets.

[27] Callison K., Ward J. (2021). Associations Between Individual Demographic Characteristics and Involuntary Health Care Delays as a Result of COVID-19: Study examines associations between individual demographic characteristics and involuntary health care delays as a result of COVID-19. Health Aff..

[28] Han B.A., Schmidt J.P., Alexander L.W., Bowden S.E., Hayman D.T., Drake J.M. (2016). Undiscovered Bat Hosts of Filoviruses. PLoS Negl. Trop. Dis..

[29] Jang B., Lee M., Kim J.W. (2019). PEACOCK: A Map-Based Multitype Infectious Disease Outbreak Information System. IEEE Access.

[30] Untergasser A., Ruijter J.M., Benes V., van den Hoff M.J. (2021). Web-based LinRegPCR: Application for the visualization and analysis of (RT)-qPCR amplification and melting data. BMC Bioinform..

[31] Liao P., Fang Y., Chen H., Wu Y., Deng Y., Chen Z., He N., Liu H., Li S. (2019). QuickAnalysis: A software designed and developed for a portable on-site pathogen detection system. J. Nanosci. Nanotechnol..

[32] Parastar H., Shaye H. (2015). MVC app: A smartphone application for performing chemometric methods. Chemom. Intell. Lab. Syst..

[33] Bustin S.A., Benes V., Garson J.A., Hellemans J., Huggett J., Kubista M., Mueller R., Nolan T., Pfaffl M.W., Shipley G.L. (2009). The MIQE Guidelines: Minimum Information for Publication of Quantitative Real-Time PCR Experiments. Clin. Chem..

